# Characterization of Male-Produced Aggregation Pheromone of the Bean Flower Thrips *Megalurothrips sjostedti* (Thysanoptera: Thripidae)

**DOI:** 10.1007/s10886-019-01054-8

**Published:** 2019-02-21

**Authors:** Saliou Niassy, Amanuel Tamiru, James G. C. Hamilton, William D. J. Kirk, Roland Mumm, Cassie Sims, Willem Jan de Kogel, Sunday Ekesi, Nguya K. Maniania, Krishnakumari Bandi, Fraser Mitchell, Sevgan Subramanian

**Affiliations:** 1 International Centre of Insect Physiology and Ecology (icipe), P.O. Box 30772-00100, Nairobi, Kenya; 2 School of Life Sciences, Keele University, Huxley Building, Staffordshire, ST5 5BG UK; 3 Infectious Disease Transmission and Biology Group, Department of Biomedical and Life Sciences, Faculty of Health and Medicine, Lancaster University, Lancaster, LA1 4YG UK; 4 Wageningen University & Research, P. O. Box 16, 6700AA Wageningen, The Netherlands

**Keywords:** Thrips pheromone, *Megalurothrips sjostedti*, Grain legumes, Olfactometer bioassay, Headspace analysis

## Abstract

Aggregation of the bean flower thrips, *Megalurothrips sjostedti* (Trybom) (Thysanoptera: Thripidae), has been observed on cowpea, *Vigna unguiculata* (L.) Walp. To understand the mechanism underpinning this behavior, we studied the responses of *M. sjostedti* to headspace volatiles from conspecifics in a four-arm olfactometer. Both male and female *M. sjostedti* were attracted to male, but not to female odor. Gas chromatography/mass spectrometry (GC/MS) analyses revealed the presence of two distinct compounds in male *M. sjostedti* headspace, namely (*R*)-lavandulyl 3-methylbutanoate (major compound) and (*R*)-lavandulol (minor compound); by contrast, both compounds were only present in trace amounts in female headspace collections. A behavioral assay using synthetic compounds showed that male *M. sjostedti* was attracted to both (*R*)-lavandulyl 3-methylbutanoate and (*R*)-lavandulol, while females responded only to (*R*)-lavandulyl 3-methylbutanoate. This is the first report of a male-produced aggregation pheromone in the genus *Megalurothrips*. The bean flower thrips is the primary pest of cowpea, which is widely grown in sub-Saharan Africa. The attraction of male and female *M. sjostedti* to these compounds offers an opportunity to develop ecologically sustainable management methods for *M. sjostedti* in Africa.

## Introduction

Cowpea, *Vigna unguiculata* (L.) Walp., is widely grown in sub-Saharan Africa for human consumption, as a source of income and livestock feed. The bean flower thrips, *Megalurothrips sjostedti* (Trybom) (Thysanoptera: Thripidae), is one of the major limitations of cowpea production in Africa, causing 21–83% yield loss due to flower abortion and reduction in quality (Alao et al. 2011; Mfuti et al. 2017; Tamò et al. 2002). Most smallholder farmers in the region cannot afford and/or get easy access to pesticides (Jackai and Adalla 1997), while other farmers spray frequently (Abtew et al. 2016), potentially leading to high pesticide residues (Akoto et al. 2013). The development of ecologically friendly management strategies for bean flower thrips could reduce reliance on pesticides on cowpea and other legumes, leading to safer food, higher yields, better safety for farm workers, reduced environmental impact and more sustainable agriculture.

Thrips display aggregation behavior primarily so as to locate mates and increase their chance of mating (Terry and Gardner 1990; Terry 1997). Male aggregation in species such as *Frankliniella occidentalis* (Pergande), *F. schultzei* (Trybom), *Thrips fuscipennis* (Haliday) and *T. major* (Uzel), takes place in the corolla of flowers (Kirk 1985; Milne et al. 2002; Terry and Gardner 1990). In contrast, *Pezothrips kellyanus* (formerly *Megalurothrips kellyanus*) forms male aggregations on leaves and ripe fruit (Webster et al. 2006). Empirical evidence has shown that aggregation is mediated by pheromones produced by male thrips (Hamilton et al. 2005). The chemical constituents of the aggregation pheromones of *F. occidentalis* (Hamilton et al. 2005)*, F. intonsa* (Trybom) (Zhang et al. 2011) and *Thrips palmi* Karny, 1925 (Akella et al. 2014) have been identified.

Recently, we observed aggregation of male bean flower thrips on leaves of cowpea, which suggested they used an aggregation pheromone (Niassy et al. 2016). This is further supported by the presence of sternal glands, implicated in production of aggregation pheromone in other thrips species, in male bean flower thrips (Krueger et al. 2015). The identification of semiochemicals mediating aggregation in thrips can contribute to the development of novel and ecologically sound approaches to control these pests (Kirk 1997, 2017; Suckling et al. 2008). For example, identification of the aggregation pheromone in the western flower thrips led to the development of commercial lures for monitoring and mass trapping (Kirk and Hamilton 2003; Sampson and Kirk 2013).

In this study, we (i) demonstrate the production of aggregation pheromone by male *M. sjostedti,* (ii) characterize components of *M. sjostedti* headspace volatiles and (iii) demonstrate the behavioral activity of bean flower thrips to authentic standards of the identified compounds. Our findings pave a way for developing ecologically friendly, pheromone-based management strategies to protect cowpea and other grain legume crops from bean flower thrips.

## Methods and Materials

### Insects

Adult *M. sjostedti* were collected from cowpea in an experimental field at *icipe*-Thomas Odhiambo Campus, Mbita, western Kenya (0°25’S, 34°12’E c. 1200 m above sea level), using the whole plant tapping method over a white tray (Pearsall and Myers 2000). Adult *M. sjostedti* were sorted into male- or female-only groups and transferred to cleaned glass vials using an aspirator. Male and female *M. sjostedti* were separated visually. Female *M. sjostedti* are shiny black with a larger body size and abdomen, while males have a brownish body color with a slender abdomen.

### Volatile Collection

Headspace volatiles were collected from male and female *M. sjostedti* using a dynamic headspace sampling technique (Tamiru et al. 2011). Prior to volatile collection, 1000 male and 1000 female *M. sjostedti* were placed separately inside cleaned screw-top glass containers (1000 ml, Borgonovo, Italy). The screw-tops, fitted with inlet and outlet ports, were cleaned with detergent and rinsed with 70% ethanol and distilled water, while the glass containers were rinsed with hexane and heated at 180 °C for 3 hr before use. Air, passed through a charcoal-filter to remove volatile contaminants, was pumped continuously (250 ml.min^−1^) through the inlet port using a portable air entrainment apparatus with an inbuilt flow meter to measure and adjust airflow. The headspace volatiles were collected for 24 hr in the laboratory (65 ± 10% RH; 25 ± 3 °C; L12: D12) on Porapak Q (0.05 g, 60/80 mesh; Supelco®) filters connected to the outlet port, through which air was actively drawn at 200 ml.min^−1^. Collections were undertaken over 24 hr to ensure that we included periods of pheromone release, which can vary with time of day. Thrips remained at the bottom of the glass jars and did not come in contact with the inlet/outlet ports at the top during headspace sampling. The transparent glass containers allowed monitoring of thrips movement. No plant material was present during volatile collection. Collected volatiles were eluted from the Porapak Q filters with 0.5 ml dichloromethane (DCM, ≥99% GC, Sigma Aldrich) into labeled sample vials (2 ml) and stored at −20 °C until used in the bioassays or chemical analyses. The entrainment was repeated four times.

### Olfactometer Bioassays with Headspace Volatiles

The behavioral responses of female and male *M. sjostedti* to conspecific odors were tested in a Perspex four-arm olfactometer as described in Tamiru et al. (2011, 2012). Air was drawn through the four arms toward the centre of the apparatus at 260 ml.min^−1^. A choice test was used to compare the responses of male or female *M. sjostedti* to headspace volatiles collected from male or female conspecifics. This was carried out by placing a test stimulus (10 μl aliquot of *M. sjostedti* headspace sample, corresponding to ca. 480 thrips x hours equivalents) in one of the arms, with the remaining three arms containing blank controls. The blank controls were collected at the same time from an identical set up without *M. sjostedti* present.

The headspace samples and solvent control (10 μl aliquots in each arm) were applied to a piece of filter paper (4 × 25 mm) using a micropipette (Drummond “microcap”, Drummond Scientific Co., Broomall, PA, USA) and placed in the inlet ports at the end of each olfactometer arm after allowing the solvent to evaporate for 30 sec. The test insects were transferred individually into the central chamber of the four-way olfactometer and observed. Observation started once the thrips started to move and was continued for 12 min (Mwando et al. 2018). An insect that remained motionless for 2 uninterrupted minutes at the start was considered inactive and discarded. Time spent by the test insect in each olfactometer arm was recorded using OLFA behavioral analysis software (Udine, Italy). The bioassay arena was illuminated by uniformly diffuse fluorescent lamp light (18 W), 60 cm above the olfactometer, and surrounded by black cloth to prevent any stray light from the surroundings. The bioassay setup was rotated every 3 min to avoid any directional bias.

### Analysis of Headspace Volatiles

Male and female *M. sjostedti* headspace volatiles were compared, and peaks that were apparent in males identified by gas chromatography coupled mass spectrometry (GC/MS), using conditions as detailed in Akella et al. (2014). Injections of the volatile samples were made on an Agilent 7890 GC/5973 MS (Agilent Technologies, Stockport, UK), using DB-5MS and DB-WAX capillary columns (both 30 m, 0.25 mm i.d., 0.25 μm film thickness, Supelco, Poole, UK). The oven temperature was maintained at 40 °C for 2 min, and then increased at 10 °C min^−1^ to 120 °C, 6 °C min^−1^ to 180 °C, and 0 °C min^−1^ to 250 °C, and held for 1 min. Ionization was performed by electron impact (70 eV, source temp. 180 °C).

The identities of the two male-produced compounds were confirmed by comparing their retention indices (RI) and mass spectra against authentic samples from a library of 200 synthetic monoterpene C5 esters (Akella et al. 2014) and associated terpene alcohols on two GC columns of different polarities (DB-5MS and DB-WAX). The library was prepared by synthesizing 200 of the possible combinations of 19 five-carbon fatty acids and their isomers with 17 commercially available acyclic, monocyclic and bicyclic monoterpene alcohols and their isomers (Akella et al. 2014). The identity and stereochemistry of each of the compounds was determined by co-injection on the GC/MS fitted with a CycloSil-B column (30 m, 0.25 mm i.d., 0.25 μm film thickness, Agilent Technologies). The GC column oven temperature was initially 50 °C and increased at 0.025 °C min^−1^ to a final temperature of 75 °C, and held for 35 min.

### Synthesis of Synthetic Pheromone Components

The (*R*) enantiomer of lavandulol was obtained from racemic lavandulol (95% purity, Sigma-Aldrich, Gillingham, UK) by a lipase-catalysed acylation using porcine pancreas lipase type II (Sigma-Aldrich) (Zada and Dunkelblum 2006). Chiral GC showed the (*R*)-lavandulol obtained had an enantiomeric excess of 97% and a purity of 99%.

The (*R*)-lavandulyl 3-methylbutanoate ester was prepared by a Steglich esterification of (*R*)-lavandulol with 3-methylbutanoic acid (99% purity, Sigma-Aldrich). The esterification method has been described in detail for the production of lavandulyl 3-methyl-3-butenoate (Akella et al. 2014). Chiral GC showed the (*R*) enantiomer to have an enantiomeric excess of 97% and a purity of 99%.

### Olfactometer Assays with Synthetic Pheromone Compounds

Synthetic standards of pheromone compounds (30 μg) identified from male *M. sjostedti* headspace samples were tested for attractiveness to male and female *M. sjostedti* in a bioassay setup similar to that for testing the headspace volatiles. A synthetic compound, loaded on slow-release rubber septa (Supelco®), was placed in one of the arms, with the remaining three arms containing blank controls (only septa). The time spent by the test insects (male or female *M. sjostedti*) in each olfactometer arm was recorded with OLFA software as described previously.

### Statistical Analysis

Statistical analysis compared behavioral responses (attraction) of bean flower thrips to headspace volatiles from male or female conspecifics as well as synthetic standards in a four-way olfactometer choice test. Bioassay data from the olfactometer were generated using OLFA software, which provides a summary of time spent by *M. sjostedti* in each of the four olfactometer arms. The time spent (attraction) data were then converted into proportions to address dependence of visiting time by thrips within the olfactometer odor fields, and then a log-ratio transformation (log10) was applied to account for the compositional nature of the proportions (Aitchison 1986; Tamiru et al. 2011). The transformed data were checked for normality and then subjected to analysis of variance to evaluate the difference between treatment and control. The analysis was implemented in R statistical software, version 3.2.3 (R Development Core Team 2015).

## Results

### Behavioral Responses of Adult *M. sjostedti* to Male and Female Odors

**Fig. 1:**
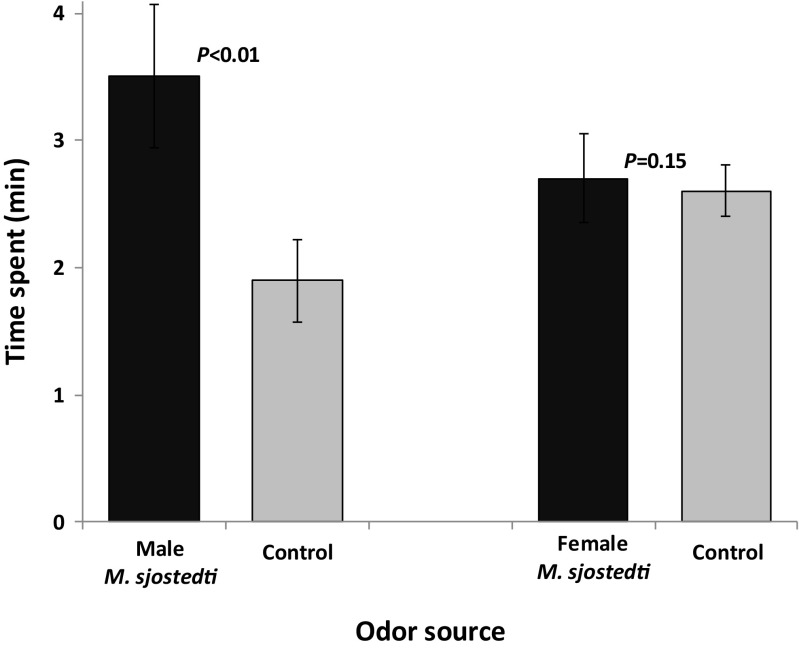
Behavioral responses of male *Megalurothrips sjostedti* to headspace volatiles collected from male or female conspecifics in a four-arm olfactometer. Each male thrips was observed for 12 min. Time spent (min; mean ± SE) by male *M. sjostedti* in the treatment and control regions of the olfactometer is shown. *P* indicates the level of significance for the difference between treatment and control

Male *M. sjostedti* were attracted to male (*F*_1,90_ = 11.79, *P* < 0.01, *N* = 23), but not to female (*F*_1, 70_ = 2.09, *P* = 0.15, *N* = 18), odor (Fig. 1).

**Fig. 2:**
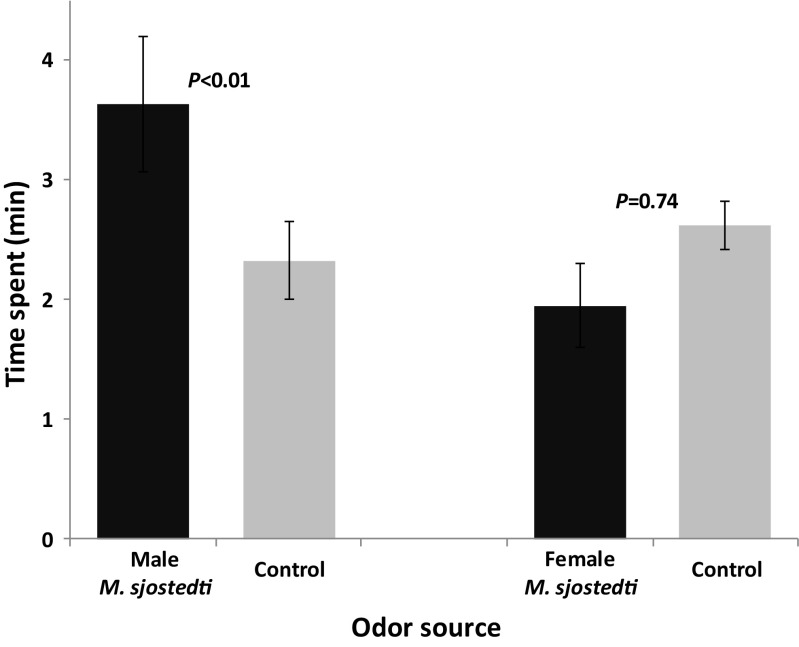
Behavioral responses of female *Megalurothrips sjostedti* to headspace volatiles from male or female conspecifics in a four-arm olfactometer. Each female thrips was observed for 12 min. Time spent (min; mean ± SE) by female *M. sjostedti* in the treatment and control regions of the olfactometer is shown. *P* indicates the level of significance for the difference between treatment and control

Female *M. sjostedti* spent more time in the arm containing male odor than in the control arm (*F*_1, 70_ = 8.59, *P* < 0.01, *N* = 18). In contrast, there was no difference in time spent by female *M. sjostedti* in the arm treated with female odor and the arm with the blank control (*F*_1, 46_ = 0.11, *P* = 0.74, *N* = 12) (Fig. 2).

### Identification of Male-Produced Compounds

**Fig. 3:**
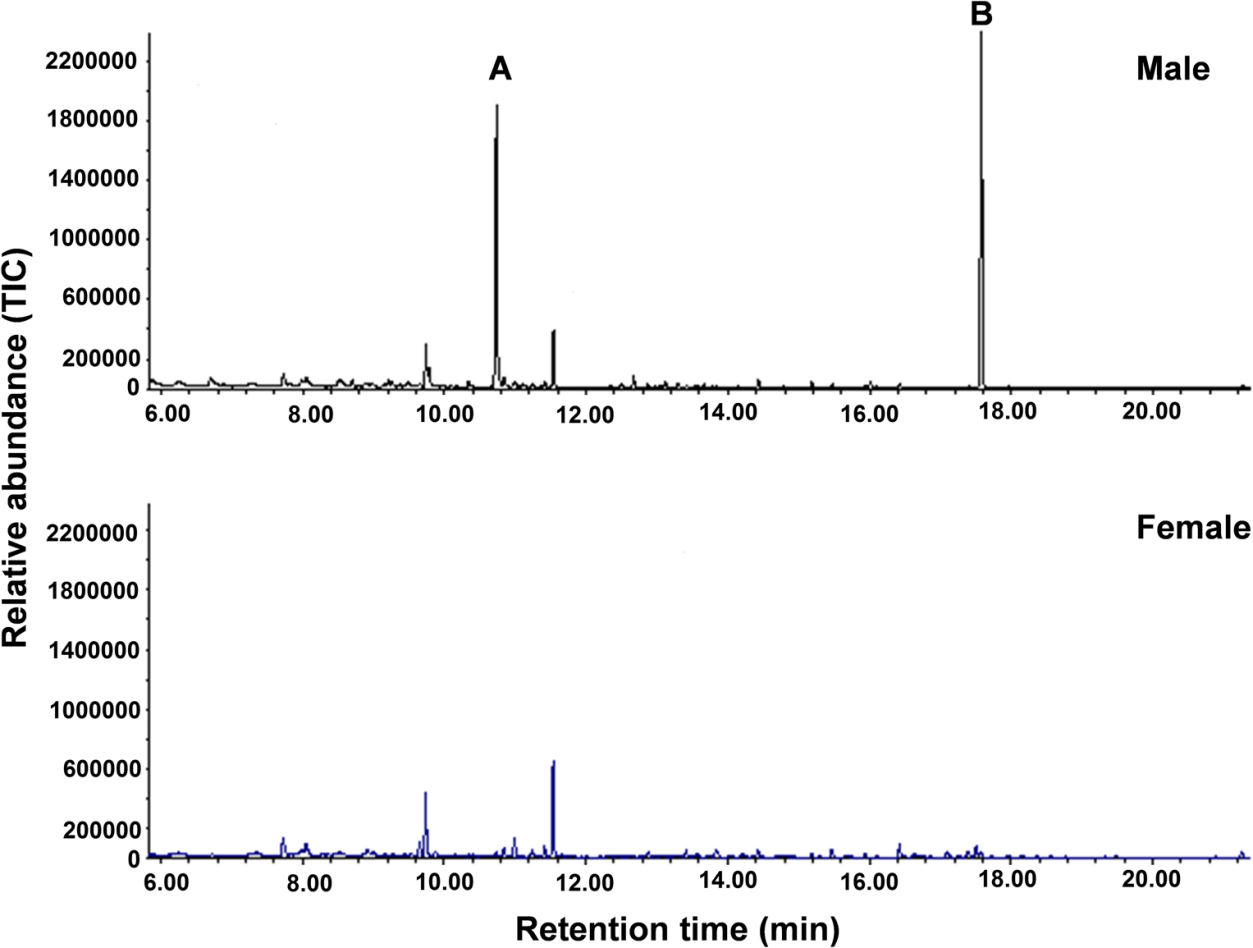
Mass chromatograms of the headspace volatiles from adult males (upper trace) and females (lower trace) of *Megalurothrips sjostedti* on a DB-5MS column. The two labeled compounds are (*R*)-lavandulol (**A**) and (*R*)-lavandulyl 3-methylbutanoate (**B**). TIC = total ion current

A detailed comparison of total ion chromatograms obtained by GC/MS analyses consistently showed that there were two distinct peaks present in collections of adult males that were present in only trace amounts in adult females (Fig. 3). The mass spectra suggested that these were a monoterpene alcohol and a monoterpene pentanoate. The mass spectrum and retention index (RI) of each of the two natural compounds were compared against those of compounds in our compound library. The two natural compounds had retention times identical with those of lavandulol and lavandulyl 3-methylbutanoate on both non-polar (DB-5MS) and polar (DB-WAX) GC columns, and the mass spectra were superimposable. However, the RIs and mass spectra of lavandulyl 3-methylbutanoate and lavandulyl 2-methylbutanoate were very similar. Therefore, the identity of the second peak was further confirmed by co-injection with an authentic standard. Lavandulyl 3-methylbutanoate gave peak enhancement on both columns, whereas lavandulyl 2-methylbutanoate did not.

**Fig. 4:**
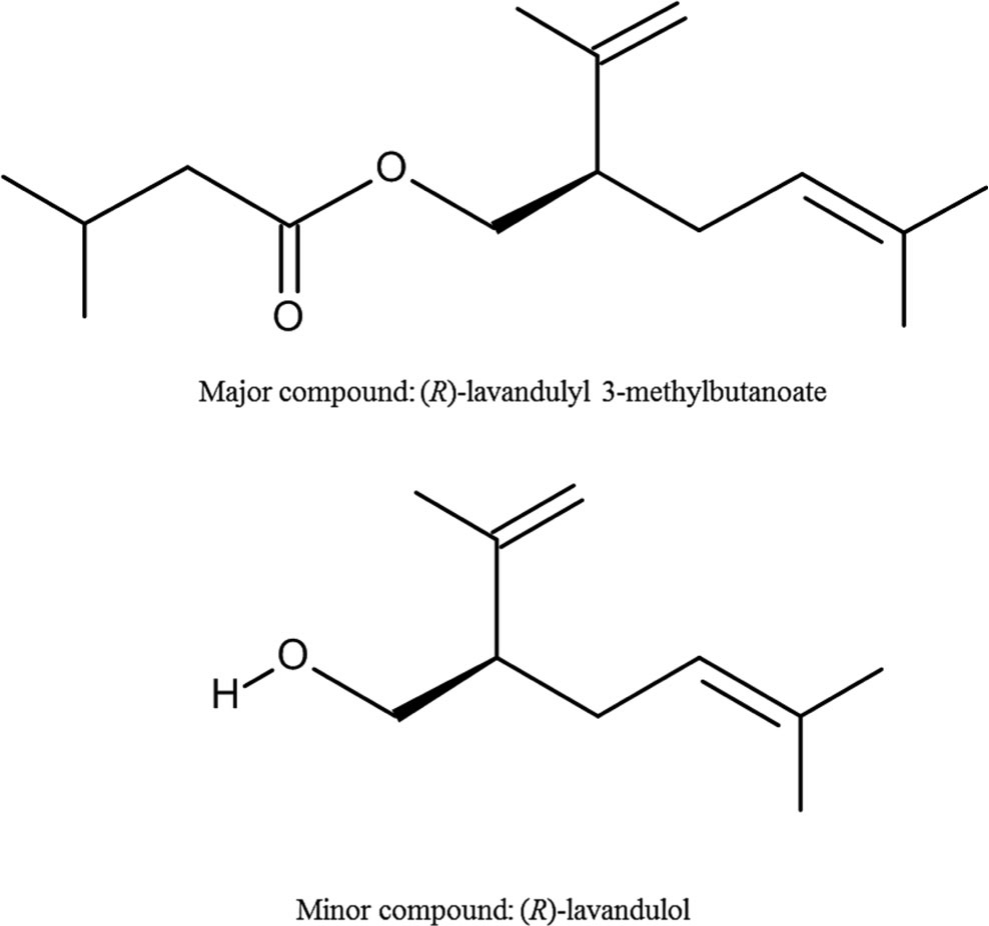
Structures of major and minor compounds of male *Megalurothrips sjostedti* aggregation pheromone

Since both of the identified compounds are chiral, the RIs were compared with authentic standards on a chiral column (CycloSil-B) and the absolute configurations confirmed by co-injection. The first (minor) peak of male *M. sjostedti* headspace was thus identified as (*R*)-lavandulol [(*R*)-5-methyl-2-(prop-1-en-2-yl)hex-4-en-1-ol; formula: C_10_H_18_O; molecular weight: 154] and the second (major) peak was identified as (*R*)-lavandulyl 3-methylbutanoate [(*R*)-5-methyl-2-(prop-1-en-2-yl)hex-4-en-1-yl 3-methylbutanoate; formula: C_15_H_26_O_2_; molecular weight: 238] (Figs. 3, 4).

### Behavioral Response of *Megalurothrips sjostedti* to Synthetic Compounds

**Fig. 5:**
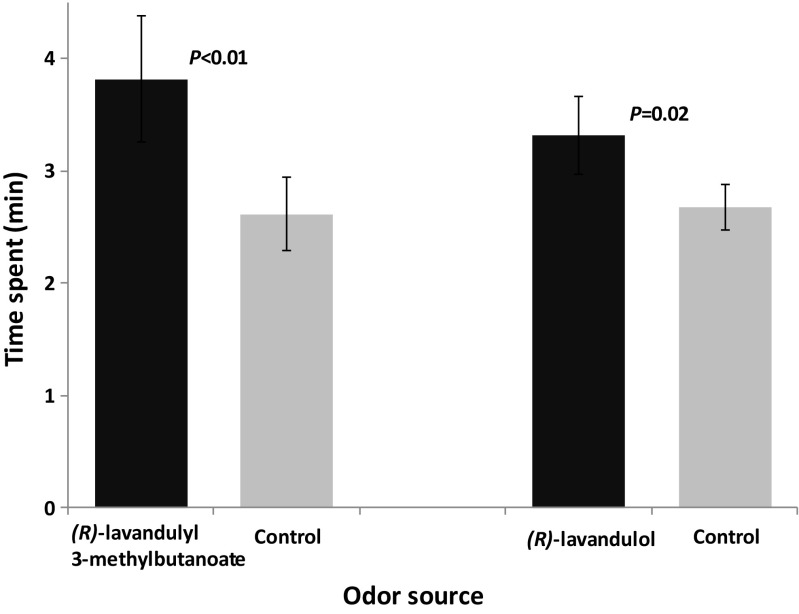
Behavioral responses of male *Megalurothrips sjostedti* to synthetic male-produced compounds in a four-arm olfactometer. Time spent (min; mean ± SE) by male *M. sjostedti* in the treatment and control regions of the olfactometer is shown. *P* indicates the level of significance for the difference between treatment and control

When synthetic versions of the two compounds were tested individually in the olfactometer, male *M. sjostedti* were attracted to each of (*R*)-lavandulyl 3-methylbutanoate (*F*_1,38_ = 8.20, *P* < 0.01, *N* = 10) and (*R*)-lavandulol (*F*_1,38_ = 6.09, *P* = 0.02, *N* = 10) (Fig. 5).

**Fig. 6:**
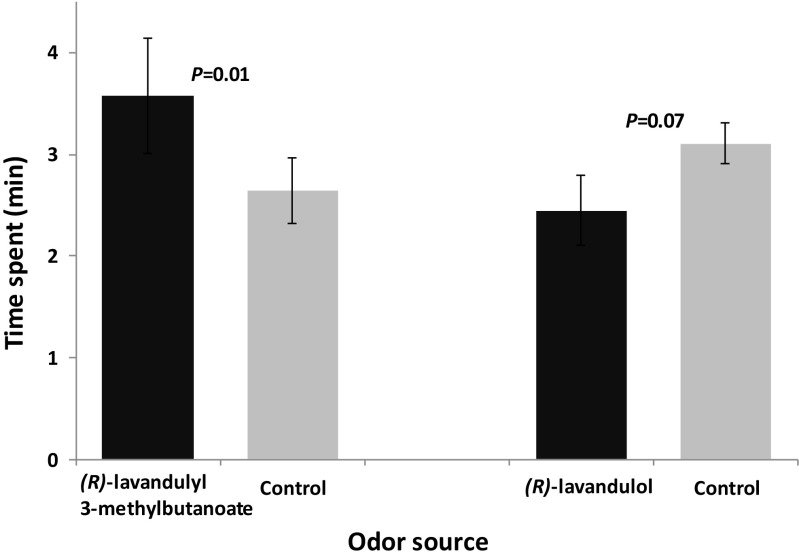
Behavioral responses of female *Megalurothrips sjostedti* to synthetic male-produced compounds in a four-arm olfactometer. Time spent (min; mean ± SE) by female *M. sjostedti* in the treatment and control regions of the olfactometer is shown. *P* indicates the level of significance for the difference between treatment and control

Female *M. sjostedti* showed preference for (*R*)-lavandulyl 3-methylbutanoate (*F*_1,38_ = 6.90, *P* = 0.01, *N* = 10) over the control, but did not show any preference for (*R*)-lavandulol (*F*_1,38_ = 3.57, *P* = 0.07, *N* = 10) compared to the control (Fig. 6).

## Discussion

The dispersion patterns of *M. sjostedti* adults in the field indicate that there is active aggregation, which implies a role for intrinsic factors such as pheromones (Niassy et al. 2016; Salifu and Hodgson 1987). Our study of the behavioral responses of *M. sjostedti* from Mbita, Kenya to conspecific odors revealed that both male and female *M. sjostedti* were attracted to headspace volatiles from males, demonstrating the presence of a male-produced aggregation pheromone. These results are similar to the behavioral responses reported in four other thrips species, *F. occidentalis*, *F. intonsa*, *T. palmi* and *P. kellyanus,* in which males and females responded to male odor (Akella et al. 2014; Kirk and Hamilton 2004; Webster et al. 2006; Zhang et al. 2011).

Characterization of the headspace volatiles of *M. sjostedti* revealed the presence of two compounds, highly abundant in males. Two compounds were also detected in *F. occidentalis* and *F. intonsa* (Hamilton et al. 2005; Zhang et al. 2011), whereas only one compound was detected in *T. palmi* (Akella et al. 2014). In the species in which two compounds have been found in the headspace, one is consistently in larger amounts, and these are hereafter referred to as the major and minor compounds, respectively. The major compound in the headspace volatiles of male *M. sjostedti*, (*R*)*-*lavandulyl 3-methylbutanoate, is unique among aggregation pheromone compounds of thrips identified so far. However, it is noteworthy that the major or only compound in the four species are all enantiomers of an ester of a monoterpene alcohol and a five-carbon acid, *i.e.* (*R*)-lavandulyl 3-methylbutanoate in *M. sjostedti*, (*R*)-lavandulyl 3-methyl-3-butenoate in *T. palmi* (Akella et al. 2014) and neryl (*S*)-2-methylbutanoate in *F. occidentalis* and *F. intonsa* (Hamilton et al. 2005; Zhang et al. 2011). The minor compound detected in *M. sjostedti*, (*R*)-lavandulol, is also unique among thrips aggregation pheromone compounds, and has structural similarities to the minor compound, (*R*)-lavandulyl acetate, found in both the *Frankliniella* species studied so far (Hamilton et al. 2005; Zhang et al. 2011). A minor compound can be hard to detect in insects as small as thrips and was detectable in *F. occidentalis* only when headspace volatiles were collected from large numbers of males (Dublon 2009; Dublon et al. 2008)*.* In our study, high numbers of males and females (1000 each) were used for collection of the volatiles, facilitating detection of the minor compound. This is the first thrips species for which the major and minor aggregation pheromone compounds have the same monoterpene moiety. Although this could suggest that the minor compound, (*R*)-lavandulol, is a precursor or a by-product of the major compound, the response by males to both compounds in the olfactometer suggests otherwise.

Olfactometer assays with synthetic standards showed that male *M. sjostedti* were attracted to the minor compound, (*R*)-lavandulol, whereas females were not. The role of the minor compound in thrips aggregation pheromones remains unclear. Characteristic species-specific ratios of major to minor compounds have been recorded in *F. occidentalis* and *F. intonsa* (Zhang et al. 2011; Zhu et al. 2012), although these ratios can change during the day (Li et al. 2017). Field trials in China have recently shown a differential catch between the two species with two different compound ratios (Li et al. 2018), suggesting the two compounds are components of one pheromone. However, field trials in Spain with *F. occidentalis*, using a range of release rates and ratios, did not find a ratio that caught more than the major compound alone (Dublon 2009; Sampson 2014). Laboratory bioassays have suggested that the minor compound in *F. occidentalis* could function as an aphrodisiac pheromone (Olaniran 2012). We are currently undertaking further work to establish the roles of different concentrations and ratios of the two compounds in *M. sjostedti* behavioral responses.

This study is the first to confirm the presence of an aggregation pheromone in *M. sjostedti*, a key pest of grain legumes in Africa. It also suggests a role for both the major and minor compounds in eliciting behavioral responses in the laboratory. Behavioral responses observed in the present study need to be further tested using other geographically distinct populations of *M. sjostedti*. The potential to enhance the attraction of both male and female thrips offers potential for the use of the aggregation pheromone as part of integrated pest management monitoring (Kirk 2017), mass-trapping (Sampson and Kirk 2013) or “lure and infect” (Mfuti et al. 2017). Further studies are being undertaken to test the effectiveness of the synthetic compounds under field conditions as well as to identify the most effective release rates and blends.
